# Long‐term indoor gunshot exposure of special police forces induces bronchitic reactions and elevated blood lead levels—The Berlin shooting range study

**DOI:** 10.1002/jcsm.13147

**Published:** 2022-12-20

**Authors:** Christian Witt, Camilla Kienast, Georg Bölke, Christina Hoffmann, Robert Roehle, Olaf Bender, Dennis Nowak, Rudolf Tauber, Hanns‐Christian Gunga, Peter Hoffmann, Andrew J.S. Coats, Uta Liebers

**Affiliations:** ^1^ Department of Outpatient Pneumology Charité – Universitätsmedizin Berlin, corporate member of Freie Universität Berlin, Humboldt‐Universität zu Berlin Berlin Germany; ^2^ Institute of Physiology, Center for Space Medicine and Extreme Environments Berlin Charité – Universitätsmedizin Berlin, corporate member of Freie Universität Berlin, Humboldt‐Universität zu Berlin Berlin Germany; ^3^ Charité Coordinating Center for Clinical Studies (KKS) Charité – Universitätsmedizin Berlin, corporate member of Freie Universität Berlin, Humboldt‐Universität zu Berlin Berlin Germany; ^4^ Institute and Clinic for Occupational, Social, and Environmental Medicine University Hospital, Ludwig Maximilian University of Munich; Comprehensive Pneumology Center (CPC) Munich, member DZL, German Center for Lung Research Munich Germany; ^5^ Institute of Laboratory Medicine, Clinical Chemistry and Pathobiochemistry Charité – Universitätsmedizin Berlin, corporate member of Freie Universität Berlin, Humboldt‐Universität zu Berlin Berlin Germany; ^6^ University of Warwick Coventry UK

**Keywords:** aerosol long‐term exposure, airway obstruction, antimony, lead, long‐term gunshot emission, lung function, manganese, military medicine, particulate matter, police officers, shooter symptoms

## Abstract

**Background:**

Gunshot emissions contain toxic elements that can harm those frequently exposed, such as police officers. Several years ago, police indoor firing ranges were closed by the Berlin municipality in response to police officer health complaints, and an investigation was launched into the possible respiratory health risks of frequent gunshot emission exposure. We, therefore, conducted an exploratory cross‐sectional study to investigate clinical and functional parameters of respiratory health as well as the burden of trace elements in policemen with long‐term high exposure to indoor gunshot emissions, compared to low‐exposure and control groups.

**Methods:**

We conducted lung function tests and collected blood and urine samples from Berlin police officers and government employees who were divided into three subject groups based on exposure to gunshot emissions: high exposure (*n* = 53), low exposure (*n* = 94) and no exposure (*n* = 76). Lung function was examined using body plethysmography. Blood and urine samples were tested via inductively coupled plasma mass spectrometry for the presence of common gunshot powder elements (antimony, lead and manganese). Exposure and symptoms were assessed using records as well as questionnaires.

**Results:**

Higher exposure was associated with more respiratory symptoms during gun shooting practice (64% vs. 21%, *P* < 0.001) compared to the low‐exposure group. Headache, cough, discoloured mucous and shortness of breath were also more common as were some other symptoms. The cough symptomatology of the high‐exposure group also persisted significantly longer (median: 0.67 vs. 0.01 days, range: 0 to 5 days, *P* = 0.029) compared to the low‐exposure group. They also showed a lower forced expiratory volume in 1 s/forced vital capacity quotient (Tiffeneau index), *P* = 0.018 between the three groups and *P* = 0.005 for the high‐exposure group, a possible marker of early, subclinical bronchial obstruction. We observed increased blood lead concentrations depending on subject's age (+1.2% per year, 95% confidence interval: 0.5–1.9%, *P* < 0.001) and cumulative gunshot exposure (+0.34% per 100 000 shots, 0.02–0.66%, *P* = 0.037).

**Conclusions:**

These first results suggest that long‐term exposure to indoor gunshot emissions induces bronchitic reactions due to repeated irritation of the airways. Higher levels of exposure lead to more negatively impacted lung function and higher blood lead levels with the possible reason that more frequent exposure may mean shorter regeneration phases for the respiratory mucous membrane. We recommend a reduction of exposure to gunshot emissions in order to decrease symptoms and avoid any—even small—deterioration in spirometry.

## Introduction

In 2015, more than 138 officers and veterans of the Berlin Police Department visited the Charité—Universitätsmedizin Berlin, Division of Outpatient Pneumology, and reported health problems during and after gun shooting practice. Most of the patients had several years of weekly gun shooting practice. Among the reported symptoms, respiratory symptoms such as chronic cough and dyspnoea were common. The Berlin municipality closed its police indoor firing ranges and launched an investigation into the possible respiratory health risks of frequent gunshot emission exposure. To investigate this further, we conducted a cross‐sectional study of police department shooters and non‐shooting employees between March 2017 and May 2018.

**Figure 1:**
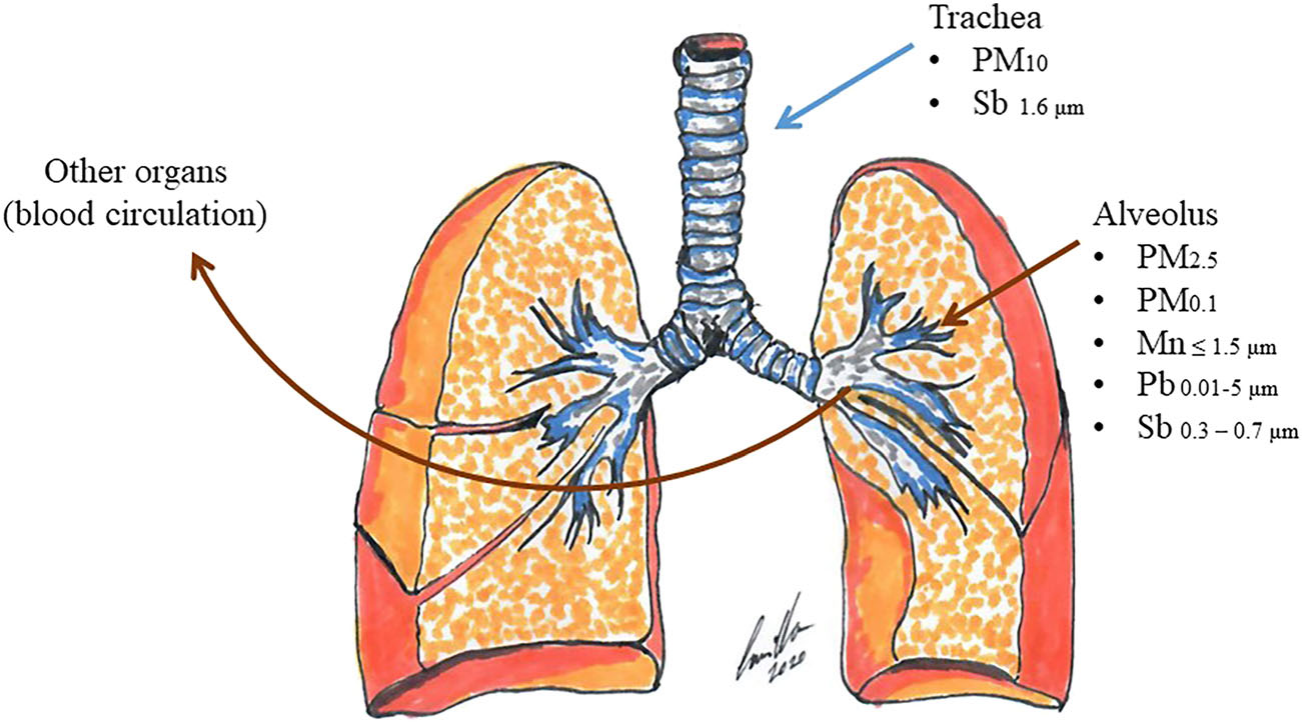
Deposition of particulate matter and trace elements in the lungs. The size and deposition site of the particulate matter (PM) including gunshot emissions in the lungs. PM 2.5–10 μm is mainly deposited on the trachea. PM < 2.5 μm can get deep into the terminal bronchioles and alveoli, and some <0.1 μm in diameter may even get into the bloodstream, thereby affecting other organs.

Past studies on the exposure to gaseous and particulate emissions from gunfire have shown that there is a health risk for shooters.^1^, ^2^, ^3^, ^4^, ^5^ Shooters, especially when firing their weapons on a regular basis, which is particularly the case for soldiers and police officers, have a greater health risk.^6^, ^7^ In addition to the obvious risks of being hit by a misdirected bullet or suffering from potential hearing damage, shooters inhale gaseous components co‐released with the gunfire during their training. This leads to an inhalational exposure to carbon monoxide, carbon dioxide, nitrous oxides, polycyclic aromatic hydrocarbons and metal particles mostly with a diameter <3.5 μm, meaning fine particulate matter (PM_2.5_) and ultrafine particulate matter (UFP) with a diameter <0.1 μm.^8^, ^9^, ^10^ Not only is the shooter exposed to these emissions but so potentially are bystanders and instructors.^11^ Depending on the type of ammunition, the emitted metal particles are mainly lead (Pb) and antimony (Sb) in leaded ammunition, or copper, zinc and manganese (Mn) in Pb‐free ammunition. These elements can be detected in blood samples 1 to 2.5 h after a training session. Prior studies have revealed correlations between gun shooting and elevated blood lead levels (BLLs), and subsequent health impacts have been investigated.^12^, ^13^, ^14^, ^15^, ^16^, ^17^, ^18^, ^19^ The effects of other combustion products and the impact on respiratory health have, however, rarely been examined. The inhalation of PM (*Figure* *1*) plays a critical role in the development of chronic lung diseases.^1^ Despite significant concentrations of gaseous and particulate emissions being inhaled during shooting, little is known about the long‐term effects of these emissions on respiratory health. In vitro studies have shown a direct toxic effect of particles emitted from Pb‐containing and Pb‐free ammunition on pulmonary epithelial cells.^2^, ^3^ In addition, these particles induce the production of proinflammatory cytokines and can promote oxidative stress. Aside from Pb, other metal particles such as Sb and Mn are emitted during shooting. Sb, in an aerosol, is thought to cause irritation of the skin, eyes and lungs and may lead to pneumoconiosis and gastrointestinal symptoms.^4^ Toxicological studies have suggested a possible neurotoxic effect for airborne Mn.^4^

Growing evidence suggests that a wide variety of respirable particles can induce lung injury and enter the blood circulation. We, therefore, designed the present study with the aim of investigating the effects of long‐term exposure to gaseous and particulate gunfire emissions on respiratory symptoms and lung function and concentrations of lead, antimony, and manganese in blood and urine samples as well.

## Methods

### Study design

This cross‐sectional study was registered with the German Clinical Trials Register (DRKS, ID DRKS00012521) and approved by the Charité—Universitätsmedizin Berlin Ethics Board (verdict EA1/002/17). All measurements and procedures complied with the Declaration of Helsinki (54th Revision 2008, Korea) regarding the treatment of human subjects. The Charité—Universitätsmedizin Berlin Data Protection Officer reviewed all questionnaires, forms and data management. Inclusion criteria were men and women between ages 25 and 65 and current non‐smokers (i.e., defined as smoking cessation for at least 3 years). Exclusion criteria were (1) current smokers and/or (2) preexisting lung disease requiring continuous medication and/or (3) renal or liver impairment.

The recruitment phase of the cross‐sectional study lasted from March 2017 to May 2018. Of 234 subjects assessed for eligibility, 223 subjects were included into the study. The participants were divided into three different subject groups: (1) subjects with no exposure to shooting ranges or gunshot exposure (*n* = 76), (2) police officers with low gunshot exposure (*n* = 94; shooting practice occurring ≤5 times a year) and (3) police officers with high gunshot exposure (*n* = 53; participating in shooting practice ≥15 times a year for at least 1 year).

We performed a cross‐sectional comparative study of police officers and government employees from Berlin and divided them into three groups on the basis of their regular shooting practice, and compared their symptoms, lung function and concentrations of Pb, Sb and Mn in blood and urine samples. To characterize the potential exposure to gunshot emissions at indoor firing ranges as a modifying factor in the effects seen in the lung function of police officers, we estimated the number of bullets each officer had shot. The members of the Berlin Police Department conduct their gunshot training at different indoor firing ranges, using various types of guns and ammunition. We, therefore, operationalized the exposure rate as a ratio between cumulative bullet shots during occupational gun shooting practice and years of exposure.

**Figure 2:**
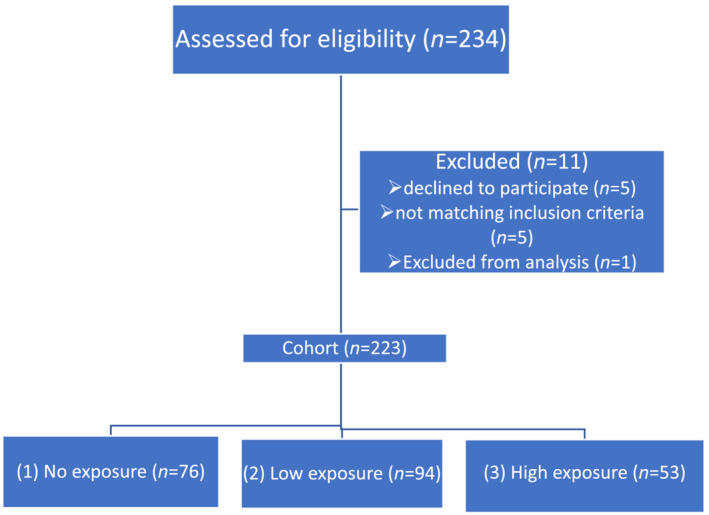
Flow diagram of subject recruitment

*Figure* *2* shows the sample size of the three groups and the number of exclusions. Group 3, with high gunshot exposure, consisted mainly of instructors and Special Task Force officers. Such officers must adhere to special requirements regarding their physical fitness and health. The calculated cumulative dose of gunshot exposure was 1000 times higher in the high‐exposure group than in the low‐exposure group. We also took into account these special requirements in selecting subjects for Groups 1 and 2 with no exposure and low exposure, respectively.

### Questionnaires

The selected subjects completed two questionnaires: The first questionnaire included demographic data, smoking history, medical history, current medication, gun shooting practice, and symptoms during and after practice at indoor firing ranges. The second questionnaire was about the duration, frequency and intensity of gunshot emission exposure. After completing the questionnaires, the study physician conducted a structured interview to ensure the correctness of the provided questionnaire answers.

### Pulmonary function tests

The lung function of each subject was analysed using a MasterScreen™ Body plethysmograph with the software JLAB 5.20.0.22 (CareFusion Germany 234 GmbH, Höchberg, Germany). The software was based on the reference values published by the European Respiratory Society in 1993.^20^ Additionally, the testing procedures complied with current guidelines.^21^ Pulse oximetry was performed using a PULOX PO‐300 (Novidion GmbH, Cologne, Germany). The following measurements were taken during pulmonary function tests:

1. forced vital capacity (FVC),
2. forced expiratory volume in 1 s (FEV1),
3. forced expiratory flow (FEF_25–75_),
4. peak expiratory flow (PEF),
5. residual volume (RV),
6. airway resistance (Raw) and
7. total lung capacity (TLC).

### Blood and urine analysis

Antimony (Sb), lead (Pb) and manganese (Mn) were analysed in whole‐blood and urine samples. Additionally, Mn concentrations were measured in blood serum.

The venous blood samples were drawn using a Safety‐Multifly® 21G needle with a long tube and assembled adapter (Sarstedt, Nümbrecht, Germany) and collected in three types of S‐Monovette® containers: one with K_3_ ethylenediaminetetraacetic acid (EDTA), another without additives and a container with liquid lithium heparin (Sarstedt, Nümbrecht, Germany). Urine samples were collected in Urine Monovettes® (Sarstedt, Nümbrecht, Germany).

To ensure that the sample collection material was free of Pb, Mn and Sb traces, specimens of each type of collection material were either rinsed (Safety‐Multifly® needle) or filled (sample tubes, cups) with purified water. Afterwards, Pb, Mn and Sb were measured in the water samples by inductively coupled plasma mass spectrometry (ICP‐MS).^22^, ^23^ We performed multiple analyses (*n* = 5) and calculated the mean rate of possible trace element residues. The mean rate of each trace element was less than the limit of determination (Pb 0.2 μg/L, Sb 0.2 μg/L and Mn 0.1 μg/L). The patients' specimens were obtained exclusively with previously analysed and approved material. The collection of specimens of each subject took place on one examination date.

The blood and urine samples were analysed for Pb, Mn and Sb concentrations using ICP‐MS by the Medizinisches Labor Bremen (Bremen, Germany) accredited by the Deutsche Akkreditierungsstelle (DAkkS). The applied reference values are listed in *Table* *S1*. The creatinine concentration in urine was determined by photometric measurement using the Jaffe method.

### Statistical analyses

Firstly, we calculated our required number of subjects to detect a sufficient effect size of ∆ = 0.210 (∆^2^ = 0.044) with a power of 80% at *α* = 0.05, using nQuery Advisor V.7 (Statistical Solutions Ltd, Cork, Ireland). A *P*‐value below or equal to 0.05 was considered statistically significant. Secondly, Q–Q plots and the Levene test were applied to analyse the preconditions for analysis of variance (ANOVA) testing. The non‐parametric Kruskal–Wallis test was used when the preconditions for ANOVA were still not met after transformation. For post hoc analyses, we used the *t* test or Dunn's multiple comparisons test.

The demographic data were descriptively analysed and tested using ANOVA, Kruskal–Wallis test or Fisher's exact test, according to distribution and variable type. The shooting exposure parameters were analysed using ANOVA or Kruskal–Wallis test. Fisher's exact test analysed the frequency of symptoms, and their mean duration was tested with the Mann–Whitney *U* test. The results of body plethysmography, pulse oximetry and laboratory diagnostics were analysed descriptively. If necessary, they were logarithmized and tested using ANOVA. We performed a multiple regression analysis with logarithmic Pb concentrations in the blood being the dependent variable.

## Results

The demographic and health status information of the recruited subjects are summarized in *Table* *1*. Between the three groups, the distribution of female and male subjects was unequal (*P* < 0.001, Fisher's exact test). The subjects in the high‐exposure group were predominantly male (96%) and significantly taller (median 182 cm, *P* < 0.001, ANOVA). The proportion of former smokers in the low‐exposure and no‐exposure groups was significantly higher than in the high‐exposure group (*P* = 0.009, Fisher's exact test). Also, the percentage of subjects taking regular medication was significantly higher in the low‐ and no‐exposure groups compared to the high‐exposure group (43%, *P* = 0.023, Fisher's exact test).

**Table 1:** Characteristics of the study population

Parameter	No exposure	Low exposure	High exposure	Total	*P*‐value
Number	76	94	53	223	
Male (%)	22 (28.9)	57 (60.6)	51 (96.2)	130 (58.3)	<0.001
Female (%)	54 (71.1)	37 (39.4)	2 (3.8)	93 (41.7)	<0.001
Age (years), median (range)	48 (25–63)	46 (27–61)	46 (29–58)	47 (25–63)	0.384
Height (cm), median (range)	170.5 (152–196)	177 (162–199)	182 (163–193)	177 (152–199)	<0.001
Body mass (kg), median (range)	76 (50–110)	80 (50–128)	85 (57–108)	80 (50–128)	<0.001
BMI (kg/m^2^), median (range)	24.4 (18.1–36.9)	25.6 (17.9–38.2)	26.1 (21.2–31.4)	25.2 (17.9–38.2)	0.382
Never smokers, *n* (%)	50 (65.8)	77 (81.9)	46 (86.8)	173 (77.6)	0.009
Former smokers, *n* (%)	26 (34.2)	17 (18.1)	7 (13.2)	50 (22.4)	0.009
Pack years, median (range)	0 (0–17)	0 (0–20)	0 (0–25)	0 (0–25)	0.008
Chronic drug therapy (%)	33 (43.4)	35 (37.2)	11 (20.8)	79 (35.4)	0.023

Abbreviation: BMI, body mass index.

The exposure to gunfire emissions of the low‐ and high‐exposure groups are detailed in *Table* *2*. The high‐exposure group fired a 1000‐fold higher number of gunshots per year than the low‐exposure group (*P* < 0.001, Mann–Whitney *U* test). The high‐exposure group fired 62 500 gunshots annually (median value). The overall gunshot exposure of the high‐exposure group was, on average, 1 764 000 gunshots. In contrast, the overall gunshot exposure of the low‐exposure group was, on average, 1616 gunshots (*P* < 0.001). The average number of years of gunshot emissions exposure was higher in the low‐exposure group than in the high‐exposure group (24 vs. 15 years, *P* < 0.001). Subjects of the low‐exposure group have worked more years on average compared to the high‐exposure group. By contrast, subjects of the high‐exposure group were members of the Special Task Force and had, therefore, more gun shooting practice annually.

**Table 2:** Exposure to gunfire emissions

Parameter	Low exposure	High exposure	*P*‐value
Total gunshots per year	62.5 (30–1500)	62 500 (11 000–368 000)	<0.001
Total gunshots over lifetime	1616 (400–4 210 800)	1 764 000 (22 000–13 200 000)	<0.001
Total years of exposure	24 (4–40)	15 (1–40)	<0.001

*Note*: Results expressed as median (range).

### Symptoms associated with shooting practice

**Figure 3:**
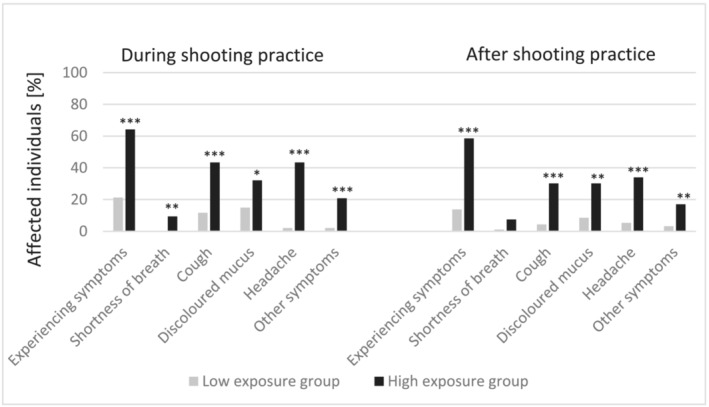
Symptoms during and after shooting practice. ^*^ *P* < 0.05, ^**^ *P* < 0.01 and ^***^ *P* < 0.001

In *Figure* *3*, we illustrate the symptoms reported by the low‐ and high‐exposure groups of police officers. The majority of subjects in the high‐exposure group showed more symptoms during (64% vs. 21%, *P* < 0.001, Fisher's exact test) and shortly after gun shooting practice (59%, *P* < 0.001, Fisher's exact test) when compared to the low‐exposure group. In both groups, subjects interrupted gun shooting practice to ‘catch their breath’, with no statistical difference found between both groups. During gun shooting practice, officers from the high‐exposure group reported more headache (43%, *P* < 0.001), cough (43%, *P* < 0.001), discoloured mucus (32%, *P* = 0.020), shortness of breath (9%, *P* = 0.005) and other symptoms (21%, *P* < 0.001). In addition to the symptoms listed in *Figure* *3*, 11 officers of the high‐exposure group reported other symptoms during gun shooting, such as irritation of eyes, skin and throat. Shortly after gun shooting practice, officers from the high‐exposure group reported headache (34%, *P* < 0.001), cough (30%, *P* < 0.001), discoloured mucus (30%, *P* = 0.001) and other symptoms (17%, *P* = 0.009). After gun shooting practice, the persistence of cough lasted significantly longer in the high‐exposure group compared to the low‐exposure group (median: 0.67 vs. 0.01 days, range: 0 to 5 days, *P* = 0.029, Mann–Whitney *U* test).

### Lung function analysis

The results of the body plethysmography analysis are shown in *Table* *3*. FEV1/FVC quotient (Tiffeneau index) differed between the three groups (*P* = 0.018, ANOVA). *t* tests showed a significant difference between the no‐ and high‐exposure groups (*P* = 0.005), as well as between the low‐ and high‐exposure groups (*P* = 0.043). The high‐exposure group had the lowest FEV1/FVC quotient (Tiffeneau index) in comparison to the other groups. The PEF increased in the subjects with no exposure up to police officers with high exposure. A post hoc analysis showed a significant difference between low‐exposure and high‐exposure groups (*P* = 0.003, Dunn's multiple comparisons test). The lung function analysis revealed higher adjusted breathing resistance (Raw) values in the no‐exposure group compared to the low‐exposure group (*P* = 0.004, *t* test) and high‐exposure group (*P* = 0.006, *t* test).

**Table 3:** Results of the physical examination

Parameter	No exposure	Low exposure	High exposure	*P*‐value
FVC (%)	103.9 (83.2–144.4)	104.1 (83.8–137.8)	103.8 (72.5–136.7)	0.897
FEV1 (%)	99.6 (72.3–139.0)	102.5 (75.8–127.0)	100.9 (64.0–132.0)	0.817
FEV1/FVC (%)	81.6 (69.9–93.4)	81.1 (61.5–91.7)	79.8 (59.8–89.0)	0.018*
PEF (%)	95.0 (70.4–140.4)	100.4 (69–132.1)	103.5 (66.5–141.3)	0.033*
FEF 75 (%)	98.0 (61.0–135.8)	97.9 (48.2–138.1)	98.8 (32.7–149.5)	0.818
FEF 50 (%)	85.1 (41.5–139.2)	89.0 (42.2–135.9)	82.1 (34.5–143.7)	0.613
FEF 25 (%)	64.4 (26.2–163.0)	64.8 (18.5–156.8)	62.1 (16.4–111.6)	0.628
Raw (%)	93.8 (37.3–148.4)	79.3 (40.7–132.1)	76.1 (41.3–161.3)	0.005**
RV (%)	122.05 (86.6–159.6)	118.7 (72.9–145.4)	121.7 (85.0–181.0)	0.274
TLC (%)	109.5 (88.4–133.9)	108.9 (86.4–129.4)	107.6 (89.7–148.0)	0.400
RV/TLC (%)	106.6 (83.8–129.1)	102.2 (75.5–127.4)	104.4 (70.2–133.9)	0.017*
Heart rate (1/min)	75 (54–108)	70 (50–100)	66.5 (49–95)	<0.001***

Abbreviations: FEF, forced expiratory flow; FEV1, forced expiratory volume in 1 s; FVC, forced vital capacity; PEF, peak expiratory flow; Raw, breathing resistance; RV, residual volume; TLC, total lung capacity.

*Note*: Results expressed as median (range).

* *P* < 0.05.

** *P* < 0.01.

*** *P* < 0.001.

The ratio between RV and TLC was within clinical reference values. Still, the no‐exposure group had a higher ratio than the exposure groups (*P* < 0.001, Dunn's multiple comparisons test). The high‐exposure group showed a significantly lower heart rate (*P* < 0.001) (*Table* *3*) and lower oxygen saturation compared to the other groups (*P* = 0.046, Kruskal–Wallis test).

### Trace element analysis

In order to examine the influence of shooting on the levels of lead, antimony and manganese in the exposed persons, the concentrations of these three elements were measured by ICP‐MS in whole blood and urine and additionally for manganese in serum in the three exposure groups. References values applied for interpretation of the concentrations of Sb, Mn and Pb in whole blood and urine and as for Mn additionally in serum as determined in the present study are summarized in *Table* *S1*. These reference values are published reference values for the general population rather than for at‐risk occupations. A selection of reference values and reference ranges from published studies and surveys without claim to completeness are given in *Table* *S2*. In particular, recent publications and reference areas were considered, which used the same ICP‐MS method as we did in the present study. As for Pb concentration in whole blood, new reference values have been published by the Federal Environmental Agency of Germany in 2018/2019 (reference in the supporting information). Because these new reference values were published after onset of the present study, they were not applied for the interpretation of the results obtained in the present study.

#### Lead (Pb)

**Figure 4:**
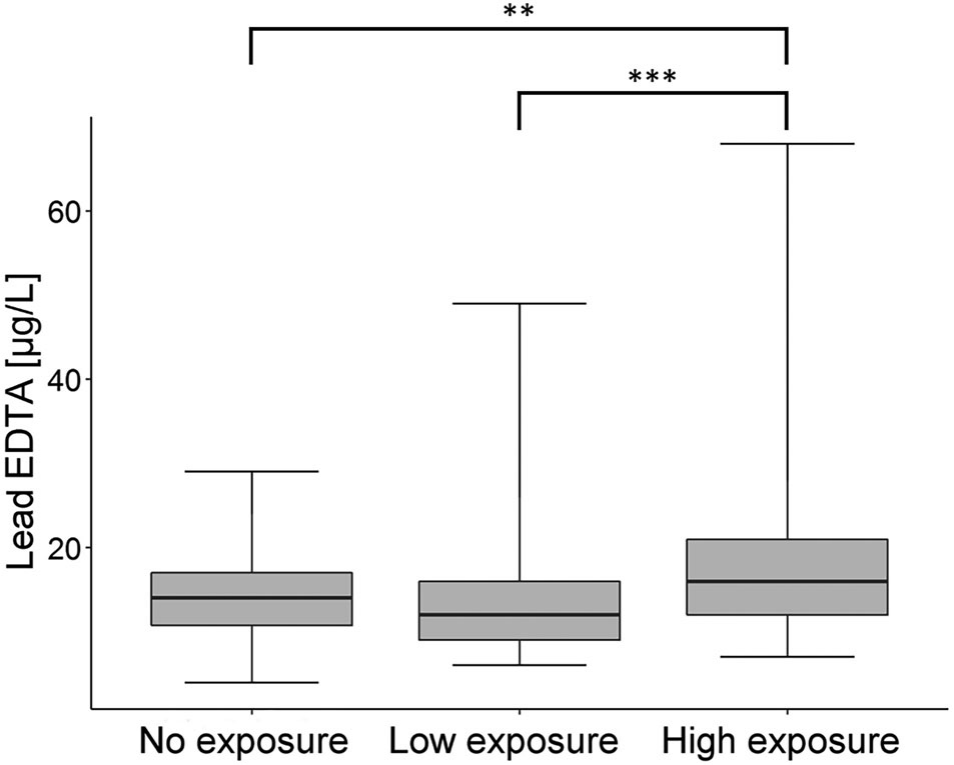
Box plot of the lead concentrations (Pb) (μg/L) in whole‐blood samples. EDTA, ethylenediaminetetraacetic acid. ^**^ *P* < 0.01 and ^***^ *P* < 0.001

The reference ranges for healthy people (see *Table* *S1*) were never exceeded. The highest blood lead levels (BLL) were measured in the high‐exposure group (*P* < 0.001, ANOVA). A multiple regression analysis was applied to investigate a possible bias by considering age, sex and smoking status. *Table* *4* summarizes the results of the multiple regression analysis. We found an increase in BLL dependent on the subject's age (1.2% per year, 95% confidence interval [CI]: 0.5–1.9%, *P* < 0.001) and exposure to gunfire emissions (0.34% per 100 000 shots, 95% CI: 0.02–0.66%, *P* = 0.037). The regression model had a coefficient of determination *R* ^2^ = 0.11, adjusted *R* ^2^ = 0.09 (*P* < 0.001). Furthermore, an interaction term between age and exposure group was included in the regression model to analyse the age dependency in more detail. A comparison of the regression models with and without interaction term showed no significant improvement (*P* = 0.891). Figure 4 shows the distribution of the measured Pb concentrations in the three groups.

**Table 4:** Multiple regression analysis of lead (Pb) concentrations in the blood

Parameter	Coefficient	95% confidence interval	Standard deviation	*P*‐value
Axis intercept	1.996	1.676–2.317	0.163	<0.001***
Exposure in 100 000 shots	0.003	0.0002–0.007	0.002	0.037*
Age	0.012	0.005–0.019	0.004	<0.001***
Sex (male)	0.074	−0.042–0.190	0.059	0.211
Former smokers	−0.036	−0.170–0.099	0.068	0.603

* *P* < 0.05.

** *P* < 0.01.

*** *P* < 0.001.

Analyses of urine samples showed that 40 study subjects had Pb concentrations below the lower limit of determination (LoD, 0.2 μg/L) (*Table* *5B*). The Pb concentrations in the remaining samples did not exceed the reference value and did not differ between the groups (Pb: *P* = 0.131, ANOVA; Pb related to creatinine: *P* = 0.298, ANOVA). In none of the subjects of all three groups did the concentration of Pb in urine exceed the upper limit of published reference ranges (<27 μg/L according to information from the Medical Laboratory in Bremen^24^; <6.40 μg/L [non‐creatinine‐related concentration] and <5.50 μg/g creatinine [creatinine‐related concentration]).^25^

#### Antimony (Sb)

The Sb concentration in whole‐blood samples was below the lower LoD of 0.2 μg/L in all three groups of our study, except for two subjects (*Tables* *5A* and *5B*). The Sb concentration in the blood of one subject of the no‐exposure group was 2.1 μg/L. The Sb concentration in the blood of one subject of the high‐exposure group was 0.2 μg/L.

**Table 5A:** Trace metal concentrations in whole blood or serum (μg/L)

	No exposure	Low exposure	High exposure	*P*‐value
Lead (Pb) (whole blood)	14 (4–29)	12 (6–49)	16 (7–68)	<0.001
Manganese (Mn) (whole blood)	7.6 (4–26.5)	6.9 (3.8–19.5)	7.5 (2.7–13.6)	0.160
Manganese (Mn) (blood serum)	0.5 (0.3–0.8)	0.5 (0.3–1.1)	0.5 (0.2–1.2)	0.158

*Note*: Results expressed as median (range).

**Table 5B:** Number of study subjects having trace metal concentrations below LoD (and % of the respective study group in brackets)

	No exposure	Low exposure	High exposure
Urine lead	19 (25)	15 (16)	6 (11.3)
Blood antimony	75 (98.7)	94 (100)	52 (98.1)
Urine antimony	76 (100)	93 (98.9)	52 (98.1)
Urine manganese	65 (85.5)	86 (91.5)	48 (90.1)

Abbreviation: LoD, limit of determination.

The results of two urine samples, one of the low‐exposure group and one of the high‐exposure group, showed values above the LoD with Sb concentrations of 0.2 μg/L. The high‐exposure group showed no increased concentrations of Sb in whole‐blood and urine samples compared to the other two groups. The concentrations of Sb in whole blood and urine in the high‐exposure group were either below or precisely at the LoD.

#### Manganese (Mn)

In the whole‐blood specimens of 12 subjects, we detected Mn concentrations above the upper reference range limit of 12.4 μg/L: in five subjects of the no‐exposure group, three subjects of the low‐exposure group and four of the high‐exposure group. There was no difference in the whole‐blood Mn concentrations between the groups (*P* = 0.160, ANOVA), as well as in the serum, Mn concentrations did not differ between the groups (*P* = 0.158, ANOVA, *Table* *5A*).

The measurements of urine Mn concentration showed that the Mn concentration in most of the study subjects was below the LoD of 0.1 μg/L (85.5% of the no‐exposure group, 91.5% of the low‐exposure group and 90.6% of the high‐exposure group) (*Tables* *5A* and *5B*). Only 11 subjects of the no‐exposure group, eight subjects of the low‐exposure group and five subjects of the high‐exposure group had Mn concentrations above the LoD. All determined urine Mn concentrations were within the reference range limits. Due to the low number of subjects with quantifiable urine Mn concentrations, no statistical test was conducted.

## Discussion

This study evaluated the effects of exposure to gunfire gaseous and particulate emissions on pulmonary health and trace metal concentrations in the body. Symptoms of the upper and lower respiratory tract, such as cough, shortness of breath and discoloured mucus, were significantly more frequent and persisted longer in the high‐exposure group. Other symptoms, such as headaches, were also found much more frequently and persistently among probands of the high‐exposure group. These persistent respiratory symptoms suggest that intensive and frequent indoor gun shooting may overburden the clearance capacity of respiratory mucosa.

### Focus on lung function results

Voie et al. showed that after 60 min of rifle shooting, 54 Norwegian men (*n* = 55) experienced headache, cough and shortness of breath.^6^ Higher annual and total gunshot emission exposure seems to be a plausible explanation for the greater incidence and longer duration of symptoms experienced by subjects of the high‐exposure group. The irritative stimulus in the respiratory tract appears to have a stronger effect. Due to higher and more frequent exposure, the regeneration phases of the mucous membrane in the respiratory tract shortens. Therefore, higher and more frequent exposition to gun shooting could trigger and increase the respiratory susceptibility to gunshot emissions, as was observed in the present study.

Median lung function values were in the normal range for all three groups, as would be expected in a healthy population with no history of lung disease. The average FVC value and FEV1 value were in the normal range for all the subjects. The FEV1/FVC quotient (Tiffeneau index) of the high‐exposure group was significantly below the value of the low‐ and no‐exposure groups. A Tiffeneau index below 70% is one of the major characteristics of obstructive lung diseases such as chronic obstructive pulmonary disease.^26^ The median Tiffeneau index of the high‐exposure group was 79.75% and, therefore, still within the reference range of values. However, when combined with the perceived symptoms of the high‐exposure group during and after gunshot exposure, this difference can be viewed as a discrete sign of pathological changes in the respiratory tract. Borander et al. reported a significant decline in lung function after shooting practice in a group of healthy, non‐smoking males employed in the Norwegian armed forces.^10^ The study showed that shooting for 60 min with an HK416 assault rifle worsened the Tiffeneau index of all the 54 subjects tested. The Norwegian study subjects had an average Tiffeneau index 90 to 150 min after shooting that was 2.0% lower than the average index of 77% before the experiment. Within 24 h after the experiment, the Tiffeneau index was 3.5% lower compared to before exposure.

Findings in other higher risk occupations^27^ differ from the results obtained in the present study. Even though a lower Tiffeneau index and persistent respiratory symptoms suggested a chronic inflammatory effect on the airways, other pulmonary function parameters did not confirm this finding. The results may be influenced by the fact that the high‐exposure group is mainly composed of Special Task Force members, who are often more athletic and healthier than the general population. The oxygen saturation determined by pulse oximetry was statistically significantly lower in the high‐exposure group compared to the other groups, but not within a pathological range. The average resting heart rate of the high‐exposure group was lower than in the other groups, which underlines the better physical condition of these subjects.

### Focus on metal levels

The reference range for healthy people for Pb of <70 μg/L for women and <90 μg/L for men^28^ was never exceeded by our subjects. *Figure* *4* shows the distribution of the measured Pb concentrations in the three groups. The highest BLL was measured in the high‐exposure group (*P* < 0.001). Regression analysis showed an increase in Pb concentration with increasing age (*P* < 0.001) and gunshot exposure (*P* = 0.037). In line with our results, a study on Italian police shooting instructors showed that the measured BLL was far below the biological limit values, but was significantly higher than in a control group without occupational shooting exposure.^29^ The authors also observed that the concentration of Pb in the blood (BLL) increased with age in the entire study population.^29^ This result indicates that high gun shooting exposure can increase in Pb concentrations in whole blood and is dependent on the frequency and duration of exposure.

Previous studies on the relationship between occupational gun shooting exposure and BLL have shown similar results. A study of Swedish police officers in 1999 showed an average BLL of 50 μg/L in male police officers and 37 μg/L in female police officers.^7^ Löfstedt et al. reported a positive correlation between BLL and the number of annually fired bullets, both during work and recreational time (*r* = 0.55; *P* = 0.001). Moreover, shooting instructors had a marginally higher BLL than police officers (58 vs. 46 μg/L).^7^

Jordakieva et al. investigated the adverse health effects of high occupational exposure to ultrafine particles in 30 Austrian police shooting instructors and 30 police officers. Inhaled metal gun emissions were mainly Pb, barium, copper and Sb. The study showed that the shooting instructors had significantly higher BLL than the control group (109.33 ± 103.63 vs. 36.24 ± 20.42 μg/L).^30^

A Belgian study compared the concentrations of several elements in blood and urine samples in two groups. One group was highly exposed to gunshot emissions and included (shooting instructors, special forces and shooting range maintenance staff); the other group served as a control group and was composed of police officers working at the administration. Although most element concentrations did not differ significantly between both groups, the subjects of the high‐exposure group had significantly higher concentrations of Sb in urine and Pb in blood samples compared to the control group.^31^ Similar to the Belgian study, the high‐exposure group in the present study showed higher BLL, still within the specified normal range. In a study by Hiller and Drexler, Pb and Sb concentrations in the blood and Sb concentrations in the urine of Bavarian police officers were examined.^32^ Of the study subjects, 11% (*n* = 4) exceeded the reference values for Sb in the urine, and 21% (*n* = 8) exceeded the reference values for Sb in the blood. Two of these individuals had both an elevated urine and blood level of Sb, and both were recreational shooters.^32^

### The potential importance of metal contamination

Pb and other heavy metals contaminate the environment of shooting ranges and their nearby surroundings.^33^ Pb is one of the most abundant heavy metals, and its toxic effects cause environmental and health problems.^34^ Exposure to Pb occurs mainly through inhalation of Pb fumes and dust. Deposition in the respiratory tract of inhaled Pb depends on particle size. About 10–60% of Pb particles inhaled by mouth with a diameter range of 0.01–5 μm are deposited in the alveolar tract. By contrast, the fraction of Pb particles inhaled through the nose is lower.^35^ Most bullet projectiles are made from Pb, but a large amount of Pb is also present in the primer, which is composed of ~35% Pb styphnate and Pb peroxide.^36^, ^37^, ^38^ Due to misalignments of the gun barrel, a portion of the Pb bullet disintegrates into fine fragments while passing through the gun.^36^ Pb particles are ejected at high pressures (18 000–20 000 psi; 124–128 MPa) from the gun barrel, along with dust and fumes originating from the primer and the bullet fragments.^36^ The shooter inhales fine Pb particulates (mainly from the primer), which constitutes the proximal exposure pathway to Pb and other gunshot residues. Fine and coarse particulates attach themselves to the shooter's hands, clothing and other surfaces from both the primer and bullet fragments. They can be inadvertently ingested, providing another Pb exposure pathway.^39^, ^40^

Inhalation of aerosol particles is known to be a pathway for heavy metals to reach the internal organs of the body. Pb aerosols represent a considerable risk factor in many industries, especially if thermally strained, melted or strained mechanically, when brushed or simply cut.^41^ Gun shooting represents a combination of both thermal and mechanical straining, resulting in considerable dermal and inhalation exposure.^42^ Numerous studies have demonstrated that gun shooting could elevate BLL 1 to 2.5 h after training.^13^, ^14^, ^17^, ^43^ Moreover, Pb is associated with several health‐related issues, such as essential tremor, hypertension, cardiovascular‐related mortality, electrocardiographic abnormalities and decreased kidney glomerular filtration rate. In women, BLLs are associated with reduced foetal growth.^44^ In vitro studies showed a direct toxic effect of particles emitted from both Pb‐containing and Pb‐free ammunition on pulmonary epithelial cells. Due to gunshot particles, the production of proinflammatory cytokines and oxidative stress increased. Sb is used as a component in both leaded and unleaded ammunition, but is also naturally present in soils and released into the environment predominantly from processes involving metal mining, smelting and refining activities.^45^ Sb is also used as a treatment against parasitic diseases such as leishmaniasis.^46^ Similarly to Pb, the absorption of Sb in the respiratory tract is influenced by particle size. Sb particles of 1.6 μm diameter are absorbed in the upper respiratory tract and cleared by mucociliary clearance a few hours later. In contrast, Sb particles with a diameter of 0.3–0.7 μm penetrate deeper into the lungs.^47^, ^48^ Sb in aerosols can worsen respiratory diseases, such as pneumoconiosis, chronic bronchitis, chronic emphysema, inactive tuberculosis, pleural adhesions and respiratory irritation (characterized by chronic coughing, wheezing and upper airway inflammation).^49^, ^50^ Urinary levels of Sb were found to be higher in patients with asthma than subjects without asthma.^51^ Furthermore, exposure to Sb particles might increase the risk of lung cancer in female subjects.^45^ One study on rats showed that 27% of female rats exposed to Sb trioxide and 25% exposed to Sb ore developed lung cancer, whereas none of the male rats in any group developed lung cancer. The authors suggest that the increased gender‐specific risk of lung cancer due to Sb inhalation is attributed to lower immunological responsiveness in female rats.^52^ Employees exposed to Sb showed lower levels of immunoglobulins G1 and E than controls,^53^ indicating impaired immune system function due to Sb exposure.

Toxicological studies on Mn have shown a possible toxic response to Mn‐containing airborne particles, suggesting that it could be an important occupational and environmental hazard.^54^, ^55^ Mn phosphate has a diameter of ≤1.5 μm and can be transported more deeply into the lungs in a similar way to PM_2_._5_.^56^ Long‐term inhalation of Mn induced cough in cold seasons, dyspnoea during exercise, and episodes of acute bronchitis in exposed employees.^57^ Moreover, Mn nanoparticles produced during the discharge of Pb‐free ammunition have demonstrable neurotoxic potential.^58^ In animals, inhaled Mn nanoparticles gain access to the brain via the respiratory system.^54^, ^59^ In adults, inhaled Mn nanoparticles access the brain directly via the olfactory nerve.^60^ One in vitro study showed that exposure to Mn increased the number of reactive oxygen species (ROS), suggesting that increased ROS levels may be involved in dopamine depletion.^61^ Additionally, Mn‐containing welding fumes have been shown to cause neurological problems.^62^ Higher levels of exposure to Mn are associated with reduced performance on memory, attention and motor function tasks in children.^63^

Lastly, our study did not show a correlation between Sb concentrations and gunshot exposure. Also, the measurements of Mn concentrations in whole blood and urine did not reveal any significant difference between the three groups. Further research, such as radiological investigation of those police officers, may reveal new insights into the impact of gunshot emissions on lung health. Computed tomography might reveal inflammation of the mucous membranes in the central as well as segment and sub‐segmental bronchi, that is, radiological bronchitic signs. Hence, our findings may underestimate the effects of long‐term gunshot exposure on pulmonary function.

### Limitations

In this investigation we did not differentiate between the kind of ammunition used and the weapon type. Some of the subjects had been practising at firing ranges for more than 40 years. During this time, ammunition has developed and changed. Another limitation of our study is the smaller number of subjects in the high‐exposure group compared to the other groups. Many potential study group subjects did not fulfil the inclusion criteria, and were excluded due to smoking and/or the other disease‐related exclusion criteria. Despite extensive efforts, 54 of the targeted 100 subjects could be recruited to participate. One of the reasons was the demanding workload of the Special Task Force officers.

Subjects in the high‐exposure group were less likely to have a family history of respiratory disease and were generally less burdened by comorbidities requiring treatment or a positive smoking history compared to the other groups. However, lung disease requiring treatment was regarded as an exclusion criterion for all groups. Therefore, we believe that the groups were sufficiently comparable from a scientific point of view with regard to the study endpoints.

### Conclusions

This study examined the effects, like bronchitic reactions following long‐term exposure of police officers to indoor gunshot emissions. Pulmonary function tests revealed a significant, but still subclinical decline in the FEV1/FVC quotient (Tiffeneau index). Subjects of the high‐exposure group more frequently reported discoloured mucus, cough, shortness of breath and headache. Although still within the reference range, a higher BLL was found in individuals highly exposed to gunshot emissions. We recommend that every effort must be made to minimize the effects of exposure to gunshot emissions, especially in occupational shooters. This study may serve as a guideline when performing regular medical checkups. Furthermore, it could serve as the basis for a longitudinal study to investigate the pulmonary effects of lifelong exposure to gunshot emissions.

## Conflicts of interest

The authors report no conflicts of interest related to the subject matter of this study.

The study was funded by the Senatsverwaltung für Inneres und Sport, Berlin, Germany. The Senate Administration was involved in the planning and the refining of recruitment strategies but not in the development of the study design, the trial realization, the data interpretation or the manuscript writing process.

## Supporting information


**Table S1.** Reference ranges and values of the analytical laboratory as determined for adults by ICP‐MS.
**Table S2.** Selected published reference ranges and values for adults as determined by ICP‐MS.
