# Supplementary material for: Long‐term indoor gunshot exposure of special police forces induces bronchitic reactions and elevated blood lead levels—The Berlin shooting range study
Source: J Cachexia Sarcopenia Muscle. 2022 Dec 20;14(1):452–63. doi: 10.1002/jcsm.13147 (PMC9891938; doi:10.1002/jcsm.13147)
Supplement: Supplementary file 1 — Table S1. Reference ranges and values of the analytical laboratory as determined for adults by ICP‐MS. Table S2. Selected published reference ranges and values for adults as determined by ICP‐MS. [file JCSM-14-452-s001.docx]

**Supplementary material**

**Supplement Table 1. Reference ranges and values of the analytical laboratory as determined for adults by ICP-MS.**

| **Trace**  **element** | **Whole blood** [μg/L] | **Serum**  [μg/L] | **Urine**  [μg/L] |
| --- | --- | --- | --- |
| Antimony | < 0.2 | - | < 0.2 |
| Manganese | 5.0 – 12.4 | 0.3 – 0.9 | < 1.9 |
| Lead | <70 (♀)  <90 (♂) | - | < 27 |

♀=female, ♂=male. Literature [g, l]

**Supplementary Table 2. Selected published reference ranges and values for adults as determined by ICP-MS**

| **Trace**  **element** | **Whole blood**  [μg/L] | **Urine**  [μg/L] | **Age range** | **Literature** |
| --- | --- | --- | --- | --- |
| Antimony | - | 4.17 | 6-88 years | [a] |
| Antimony | 0.013 – 0.040 | 0.18 | 18-70 years | [b, k] |
| Antimony | 0.05 – 0.13 | 0.02 – 0.08 | - | [c] |
| Antimony | - | 0.236 | > 18 years | [d] |
| Antimony |  | 0.18 |  | [j] |
|  |  |  |  |  |
| Antimony | - | 0.17 | 3-79 years | [e] |
| Manganese | - | 3.33 | ? | [a] |
| Manganese | 5.7 – 14.6 | 0.21 | 18-70 years | [b, k] |
| Manganese | 5.0 – 12.8 | 0.11 – 1.32 | - | [c] |
| Manganese | 14.8 (♀)  14.1 (♂) | - | 18-65 years | [f] |
| Manganese | 16 (♀)  14 (♂) | - | 20-79 years | [e] |
| Lead | - | 6.40 | ? | [a] |
| Lead | 8 – 47 | 2.1 | 18-70 years | [b, k] |
| Lead | 11.4 – 62.8 | 0.01 – 2.14 | - | [c] |
| Lead | 70 (♀)  90 (♂)  30 (♀)   40 (♂) | - | 18-69 years | [g, h]  [i] |
| Lead | - | 2.81 | > 18 years | [d] |
| Lead | - | 1.9 | 20-79 years | [e] |

♀=female, ♂=male


**Supplementary Literature**

1. Paschal DC, Ting BG, Morrow JC, et al. Trace metals in urine of United States residents: reference range concentrations. Environmental research 1998; 76(1): 53-9.
2. Heitland P, Koster HD. Biomonitoring of 37 trace elements in blood samples from inhabitants of northern Germany by ICP-MS. Journal of trace elements in medicine and biology: organ of the Society for Minerals and Trace Elements (GMS) 2006; 20(4): 253-62.
3. Goulle JP, Mahieu L, Castermant J, et al. Metal and metalloid multi-elementary ICP-MS validation in whole blood, plasma, urine and hair. Reference values. Forensic science international 2005; 153(1): 39-44.
4. Hoet P, Jacquerye C, Deumer G, *et al.* Reference values and upper reference limits for 26 trace elements in the urine of adults living in Belgium. *Clin Chem Lab Med* 2013;**51**:839–49. doi:10.1515/cclm-2012-0688
5. Saravanabhavan G, Werry K, Walker M, *et al.* Human biomonitoring reference values for metals and trace elements in blood and urine derived from the Canadian Health Measures Survey 2007-2013. *Int J Hyg Environ Health* 2017; **220**:189–200. doi:10.1016/j.ijheh.2016.10.006
6. Alimonti A, Bocca B, Mattei D, *et al.* Programme for biomonitoring the Italian population exposure (PROBE): internal dose of metals. *Rapp ISTISAN* 2011; 1–85.
7. Bundesgesundheitsblatt. Aktualisierung der Referenzwerte für Blei, Cadmium und Quecksilber im Blut und im Urin von Erwachsenen. Bundesgesundheitsblatt - Gesundheitsforschung -Gesundheitsschutz 2003; 46(12): 1112-3
8. Wilhelm M, Ewers U, Schulz C. Revised and new reference values for some trace elements in blood and urine for human biomonitoring in environmental medicine. International journal of hygiene and environmental health 2004; 207(1): 69-73.
9. Bundesgesundheitsblatt. Aktualisierung der Referenzwerte für Blei im Blut von Erwachsenen. Bundesgesundheitsbl 2019; 62:1280–1284. https:// doi.org/ 10.1007/ s00103- 019- 03002-z
10. Nisse C, Tagne-Fotso R, Howsam M; Members of Health Examination Centres of the Nord Pas-de-Calais region network, Richeval C, Labat L, Leroyer A: Blood and urinary levels of metals and metalloids in the general adult population of Northern France: The IMEPOGE study, 2008–2010. Int J Hyg Environ Health 2017; 220: 341–363.
11. Heitland P, Köster H (2006b): Biomonitoring of 30 trace elements in urine of children and adults by ICP-MS. Clin Chim Acta 2006; 365: 310–318.
12. *Arbeits- und Umweltmedizinische Analysen*. 12. Auflage. Bremen, Deutschland: Medizinisches Labor Bremen 2014.
